# Optimized workflow for behavior-coupled fiber photometry experiment: improved data navigation and accessibility

**DOI:** 10.3389/fnins.2025.1601127

**Published:** 2025-07-10

**Authors:** Anna Athanassi, Amaury François, Emmanuel Bourinet, Marc Thevenet, Nathalie Mandairon

**Affiliations:** ^1^CNRS, UMR 5292, INSERM, U1028, Lyon Neuroscience Research Center, Neuroplasticity and Neuropathology of Olfactory Perception Team, University of Lyon, Lyon, France; ^2^CNRS, UMR5203, INSERM, Institut of Functional Genomics, University of Montpellier, Montpellier, France

**Keywords:** fiber photometry, video recording, behavior, olfactory perception, mice

## Abstract

Fiber photometry provides crucial insights into cell population activity underlying behavior. While numerous open-source data analysis tools exist, few offer an automated workflow that streamlines the analysis of fiber photometry data alongside behavioral measurements, by enabling more intuitive and facilitated navigation within data files. We developed here a workflow starting from the intracerebral implantation of optical fibers in mice, to the analysis of fiber photometry signals, aligned with recorded behavioral data. This tool is particularly valuable for studying unpredictable exploratory behaviors, as it allows efficient and rapid revisiting of fiber photometry signals aligned to spontaneous behavioral changes. Our approach allows ease of data analysis and exploration using custom algorithms and scripts that extract and process both fiber photometry and behavioral data, without relying on predefined event markers. We validated our method by assessing calcium activity and dopaminergic dynamics in the olfactory tubercle in response to spontaneous investigation of attractive and non-attractive odorants in freely moving adult C57BL/ 6J mice. Using jRGECO1a and dLight1.2 biosensors, we revealed distinct dopamine responses to attractive versus unattractive odorants while calcium transmission remained similar. Overall, our method significantly enhances the accessibility and efficiency of data analysis, allowing for rapid retrieval and exploration of key behavioral time points. Its adaptability makes it suitable for a wide range of spontaneous behaviors, paradigms, and sensory modalities, facilitating deeper insights into complex neural dynamics.

## 1 Introduction

Fiber photometry is a key technique for characterizing brain-behavior relationships *in vivo*, offering detailed insights into how specific brain regions contribute to behavior (Gunaydin et al., 2014; Guo et al., 2015; Martianova et al., 2019; Simpson et al., 2024; Wang et al., 2021). It involves the insertion of a fiber optic probe into the brain to detect fluorescent signals from genetically encoded biosensors. Over the past two decades, a variety of genetically encoded biosensors have been developed to study different forms of signals in the central nervous system, including neuronal and non-neuronal population activity in both superficial and deep brain structures of freely behaving animals (Abdelfattah et al., 2022; Bruno et al., 2021). For neural activity recording, calcium-sensitive sensors like GCaMP are the most widely used (Wang et al., 2021; Wu et al., 2025) but engineered genetically encoded voltage indicators (GEVIs) have also been employed (Gong et al., 2015; Kannan et al., 2018). The development of an extensive range of fluorescent sensors including dLight or GRABDA for dopamine (Patriarchi et al., 2018; Sun et al., 2018), nLightG/R for noradrenaline, GACh for acetylcholine, PsychLight2 for serotonin and Mtria for oxytocin among others, have advanced the field (Feng et al., 2024; Simpson et al., 2024). In all cases, the fiber optic probe collects emitted light, enabling real-time quantification of neural activity or neurotransmitter release. Thus, this technique provides valuable insights into cell population activity in freely moving animals, with a temporal resolution ranging from sub-second to second timescales (Murphy et al., 2023; Patel et al., 2020; Terstege et al., 2023; Wang et al., 2021). This technique has been used to better understand brain functions, by revealing the neural dynamics of sleep phases (Andersen et al., 2023; Kjaerby et al., 2022), by demonstrating how chronic stress induces anxiety-like phenotypes through changes in dopaminergic neuron activity (Morel et al., 2022), by identifying a stress susceptibility signature linked to calcium transients in the nucleus accumbens (Muir et al., 2018), or by showing modulations in calcium signaling within cortico-amygdala connections during altruistic versus selfish social choices (Scheggia et al., 2022).

Many fiber photometry studies rely on experimental paradigms designed to elicit strong and predictable behavioral and neural responses, facilitating straightforward signal-behavior correlations. These include fear conditioning (Demaestri et al., 2024; Legaria et al., 2022; Ren et al., 2018; Verharen et al., 2020; Xu et al., 2019), chronic stress models such as noise exposure (Peng et al., 2023) or social defeat (Muir et al., 2018), restraint stress (Klenowski et al., 2023), and paradigms involving explicit reinforcement such, reward learning (Sych et al., 2019) or self-stimulation (Harada et al., 2021). While these approaches provide robust, time-locked neural signatures, they impose experimenter-defined behavioral changes. In contrast, studies examining fiber photometry signals in spontaneous behavior -such as sleep cycles (Patel et al., 2020), social interaction (Gunaydin et al., 2014; Terstege et al., 2023), maternal care (Dvorkin and Shea, 2022), and spatial exploration (Loewke et al., 2021)- face a critical challenge: some behavioral events cannot be externally controlled and determined in advance based on fixed criteria. In such cases, we need the ability to dynamically reassess data, explore novel behavioral categories, or refine neural-behavioral correlations as new insights emerge. Our approach directly addresses this limitation by enabling refined data navigation, classification and retrospective behavioral segmentation. This is particularly relevant in sensory perception studies, where self-paced movements -such as in spontaneous olfactory preference paradigm- require high analytical flexibility (Gray et al., 2021). By integrating an adaptive methodology for data navigation into classified events, we offer a framework to refine the analysis of neural activity in complex, unconstrained behavioral contexts, unlocking new possibilities for studying sensory-driven decision-making and cognitive processes. Among the previously cited studies, only one (Terstege et al., 2023) provides a full script of their analysis (similar to ours) but we go further by sharing our full pipeline, including the data navigation interface of self-paced behavior. Additionally, this workflow enables the simultaneous analysis of two biosensors -for dopamine and calcium- streamlining and accelerating data processing.

The efficiency of this workflow has been demonstrated here by assessing neural activity and dopaminergic dynamics in response to olfactory stimuli. Previous research has shown that odorants with positive hedonic value compared to negative ones, can generate attraction thanks to an increased activity in the olfactory bulb (OB), the first cortical relay for olfactory information, and its target, the olfactory tubercle (OT). The OT, part of the ventral striatum, receives dopaminergic projections from the ventral tegmental area (VTA) (Midroit et al., 2021; Zhang et al., 2017). In addition, increased activity in the VTA has been observed, suggesting dopamine release in the OT in response to attractive odorants (Murata, 2020; Murata et al., 2015, 2024). Based on these findings, we thus proposed using fiber photometry to analyze neural activity and dopamine release in the OT, using, respectively, sensitive red protein calcium indicator jRGECO1a and green fluorescent genetically encoded dopamine sensor dLight1.2 (Patriarchi et al., 2018), in response to attractive versus unattractive odorants. Specifically, we injected a mixture of AAV viruses expressing jRGECO1a and dLight1.2, followed by the implantation of optical fibers in the OT of adult male mice. This enabled the detection of fluorescence while the mice freely explored a board containing an odorant source. Mice were video-tracked during fiber photometry recordings, and their exploratory behavior was segmented and referenced *post hoc* into events of interest based on the animal’s behavior (i.e., periods spent exploring the odorant source, “*olfactory investigation events”*). We developed an adaptive workflow enabling the analysis of dopamine and calcium biosensor fluctuations during selected events and found a higher level of dopamine discharge in the OT in response to attractive odorants compared to unattractive ones. Overall, this approach not only enables real-time analysis of signals during expected task events, but also allows additional behavioral events to be identified and segmented, providing a retrospective framework. This flexibility enables analyses to be refined without the constraints of predefined categories, thereby extending the applicability of this method to a wide range of experimental paradigms. Unlike built-in solutions provided by commercial systems or already existing open-source ones, this method facilitates the navigation into data files, which remains a key limitation of current existing analysis.

## 2 Materials and equipment

This section ensures full reproducibility of the experimental workflow by detailing the essential materials, equipment, and reagents required.

- Adeno-associated viruses (AAVs) for sensor expression: pAAV.Syn.NES-jRGECO1a.WPRE.SV40 (Addgene, #100854-AAV9, biosensor for calcium imaging), pAAV9-hSyn-dLight1.2 (Addgene, #111068-AAV5, biosensor for dopamine imaging).
- Surgical materials: Stereotaxic frame and nanoliter 2020 injector (World Precise Instrument WPI), isoflurane (anesthetic), lidocaine (local anesthetic), sterile saline (0.9% NaCl), stereotaxic microdrill (RWD®), microsurgical tools, resorbable suture thread (Vycril®), dental cement (CAP) and glue (Metabond), heating lamp (to maintain body temperature after surgery).
- Optical fibers (MFC_400/430–0.66_4.7mm_MF1.25_FLT, Mono Fiberoptic Cannula, Doric Lenses®).
- Fiber Photometry Systems from Doric Lenses®: LED light sources (465 nm: LEDC1-B_FC, 405 nm: LEDC1-405_FC, 565 nm LED Driver, 4 Channel Model), Fluorescence MiniCube (ilFMC6-G2_IE (410–420)_E1 (460–490)_F1 (500–540)_E2 (555–570)_F2 (580–680)_S, 6 ports with 2 integrated photodetectors and 3 integrated LEDs), patch cords (low autofluorescence, 400 μm core), rotary joint (AFRJ_2×2_PT_400–0.57 Assisted 2×2 Fiber-optic Rotary Joint, 400 μm NA0.57 fiber, 1 m input, optimized for fiber photometry).
- Behavioral apparatus: Custom-made olfactory preference test chamber and video tracking system, monomolecular odorants [Limonene 0.2% (Lim; 5,989-27-5), Citronellol 21.65% (Citro; 106–22-9), Camphor 0.46% (Cam; 76–22-2), Guaiacol 2.12% (Gua; 90–05-1), p-Cresol 1.82% (Cre; 106–44-5), and Pyridine 0.02% (Pyr; 110–86-1)] diluted in mineral oil, cotton pad (Kermen et al., 2016; Mandairon et al., 2009; Midroit et al., 2021).
- Computational tools: Data acquisition software (Doric Neuroscience Studio® (DNS V6.3.2.0)), custom MATLAB scripts for signal processing and behavioral segmentation, statistical analysis software (RStudio®), lab-made tracking software A2V Volcano.

## 3 Methods

### 3.1 Animals

Six adult (2 months old) C57BL/6 J mice were bred in house, had *ad libitum* access to food and water, and were housed on a 12 h:12 h light cycle. All experiments were done in accordance with the European Community Council Directive of 22nd September 2010 (2010/63/UE) and the National Ethics Committee (Agreement APAFIS 11725–2,017,100,915,448,101). Mice were housed in a temperature and humidity-controlled environment kept at a constant temperature of 22°C ± 2. Throughout these experiments, all efforts were made to minimize pain and discomfort of the mice.

### 3.2 Surgery

**Figure 1:**
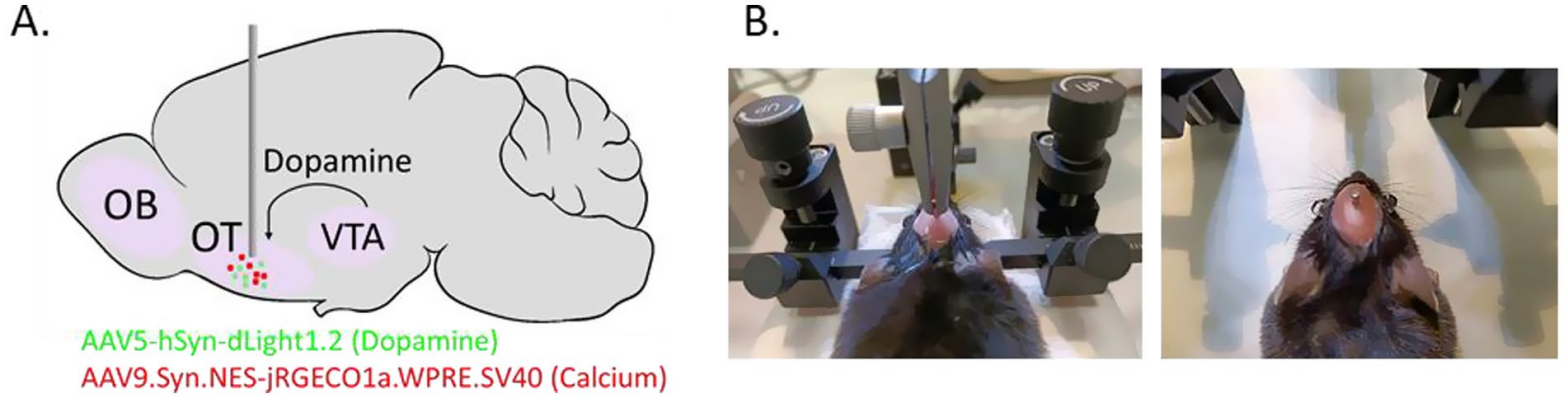
Viral injection and fiber implantation in the medial olfactory tubercle (mOT). **(A)** Two viruses, pAAV-hSyn-dLight1.2 (dopamine biosensor, 4x10e12 vg/mL) and pAAV.Syn.NES-jRGECO1a.WPRE.SV40 (calcium biosensor, 1x10e13 vg/mL) were injected in the mOT (with respect to the bregma: AP, +1.7 mm; ML, −0.5 mm; DV, −4.7 mm, 200 nL at 50 nL/min). **(B)** Photos showing the mouse position on the stereotaxic frame and the fiber implantation.

The animal was sedated using isoflurane inhalation (2.5% isoflurane 0.5% oxygen, 0.5% compressed air), and administered subcutaneous injections of Buprémorphine (0.1 mg/kg) and Metacam (20 mg/kg). Before incising the skin, local application of 2% lidocaine was administered at the incision site. The animal was then placed on a stereotaxic frame, and once securely positioned (Figure 1), the mouse’s eyes were protected with Lacrigel to prevent any ocular damage. The mouse’s scalp was sanitized alternately with ethanol and Betadine two to three times. The stereotaxic coordinates (anteroposterior (AP), lateromedial (LM)) were then set in relation to the bregma, and the horizontal alignment of the skull was confirmed by ensuring that the distance between bregma and lambda measured AP = 4.2 ± 0.2 mm. A 1.5 cm incision was made from between the eyes to the back of the head using a scalpel, and the skin edges were retracted to create an optimal field of view. The skull was kept moist with periodic drops of NaCl solution.

### 3.3 Virus injection

The two viruses, pAAV-hSyn-dLight1.2 (Addgene, #111068-AAV5, dopamine biosensor, 4x10e12 vg/mL) and pAAV. Syn. NES-jRGECO1a. WPRE. SV40 (Addgene, #100854-AAV9, calcium biosensor, 1x10e13 vg/mL) were mixed by going back and forth with the pipette and prepared for each animal just before brain injection (stock kept on ice). A glass pipette was filled with 200 nL of the mixture. Skull was pierced (0.5 mm diameter hole) over the right medial OT (with respect to the bregma: AP, +1.7 mm; ML, −0.5 mm). To avoid tissue damage, the needle was lowered into the brain trough the pre-pierced hole very slowly (0.1 μm/ 3 s) until it reached the coordinate of DV, −4.7 mm (Figure 1). The virus mixture was then injected at 50 nL/min. After a 10-min delay to ensure proper diffusion of the virus mixture in the targeted region, the needle was slowly withdrawn at the same speed (0.1 μm/3 s). To prevent blood clotting as the needle neared the scalp, the withdrawal speed was increased (0.1 μm/s). Once the pipette was removed from the brain, we sutured the skin. Between each mouse, a small amount of the mixture (50 nL) was ejected to prevent the needle from clogging. The syringe therefore had to be filled with slightly more virus than the amount injected.

### 3.4 Fiber implantation

The optical fibers were implanted 6 weeks after virus injection. Animals were placed on the stereotaxic frame using the same protocol as previously described. To enhance cement adhesion, minor scratches were made on the skull using the back of a scalpel. Using a cannula holder, an optical fiber (MFC_400/430–0.66_4.7mm_MF1.25_FLT, Mono Fiberoptic Cannula, Doric Lenses®) was implanted into the OT at the same coordinates as the viral infusion (with respect to the bregma: AP + 1.7 mm, LM − 0.5 mm, dorsoventral (DV) -4.7 mm). Given the fiber’s larger diameter compared to the infusion needle, it was lowered gradually at a rate of 0.1 mm per 10 s. To ensure optimum tissue distribution around the fiber tip, the latter was positioned 0.1 mm below the target for 1 min before being raised by 0.1 mm. One drop of glue (Metabond) was applied to the skull around the fiber to ensure stabilization before securing it with dental cement on the scalp (Figure 1). Lacrigel was reapplied to the mouse’s eyes. The mouse was then removed from the stereotaxic frame and placed in an individual cage under a heating lamp until fully awake. Mice were returned to their home cage the following day to prevent social isolation. For both surgeries, during the 5 post-operative days, the animals received daily injections of Metacam (20 mg/kg), and their weight was monitored.

### 3.5 Fiber photometry experiment combined with video recordings in an olfactory preference task

#### 3.5.1 Olfactory preference task

Mice underwent a 6-week recovery period following surgery. Recordings were conducted during an olfactory preference test, where freely moving mice explored a one-hole board apparatus during a 2-min trial (Kermen et al., 2016; Mandairon et al., 2009). During the week leading up to the test, three 2-min trials were performed in which mice explored the one-hole board apparatus without odorants (Mandairon et al., 2009) while connected to the fiber photometry setup for habituation. This also ensured that the setup allows for normal locomotor activity when the fiber is attached to the patch cord with a ceramic sleeve. Then, as a measure of odor hedonics, we measured the investigation time of six unfamiliar odorants with no known biological significance. To ensure similar perceived intensity, all odorants were diluted into mineral oil to 1 Pa (similar vapor pressure): Limonene 0.2% (Lim; 5,989-27-5), Citronellol 21.65% (Citro; 106–22-9), Camphor 0.46% (Cam; 76–22-2), Guaiacol 2.12% (Gua; 90–05-1), p-Cresol 1.82% (Cre; 106–44-5), and Pyridine 0.02% (Pyr; 110–86-1). Odor preference was defined by approach and avoidance behaviors (Kermen et al., 2016; Mandairon et al., 2009). A 60 μL of the 1 Pa odorant was applied to a small cotton square, which was placed at the bottom of a pot covered with a grid and bedding. The pot was positioned in the central hole of the board. The experiment was conducted in a dimly lit room. Before their passage, mice were kept in a separate room to prevent prior exposure to the odorant and reduce the stress associated with the experiment. Prior to each experimental trial, the mouse was connected to the setup and recorded for 2 min while in its home cage. This period reduces stress, facilitates the balance of sensor expression while controlling for daily variations in recordings, light power consistency, and fiber-optic connectivity (e.g., avoiding gaps between the patch cord, sleeve, and rotary connector). For each experimental trial, the mouse was first placed in the bottom right corner of the board and then allowed to explore freely. After the trial, the mouse was returned to its cage, the odorant pot was removed, and the board was cleaned with ethanol and allowed to dry. The order of odorant presentation was randomized across mice and only one odorant was presented per day. Each mouse was tested for the 6 odorants. For each mouse and odorant, exploration time of the hole was measured as an index of odor hedonics.

#### 3.5.2 Fiber photometry system coupled with video recording

**Figure 2:**
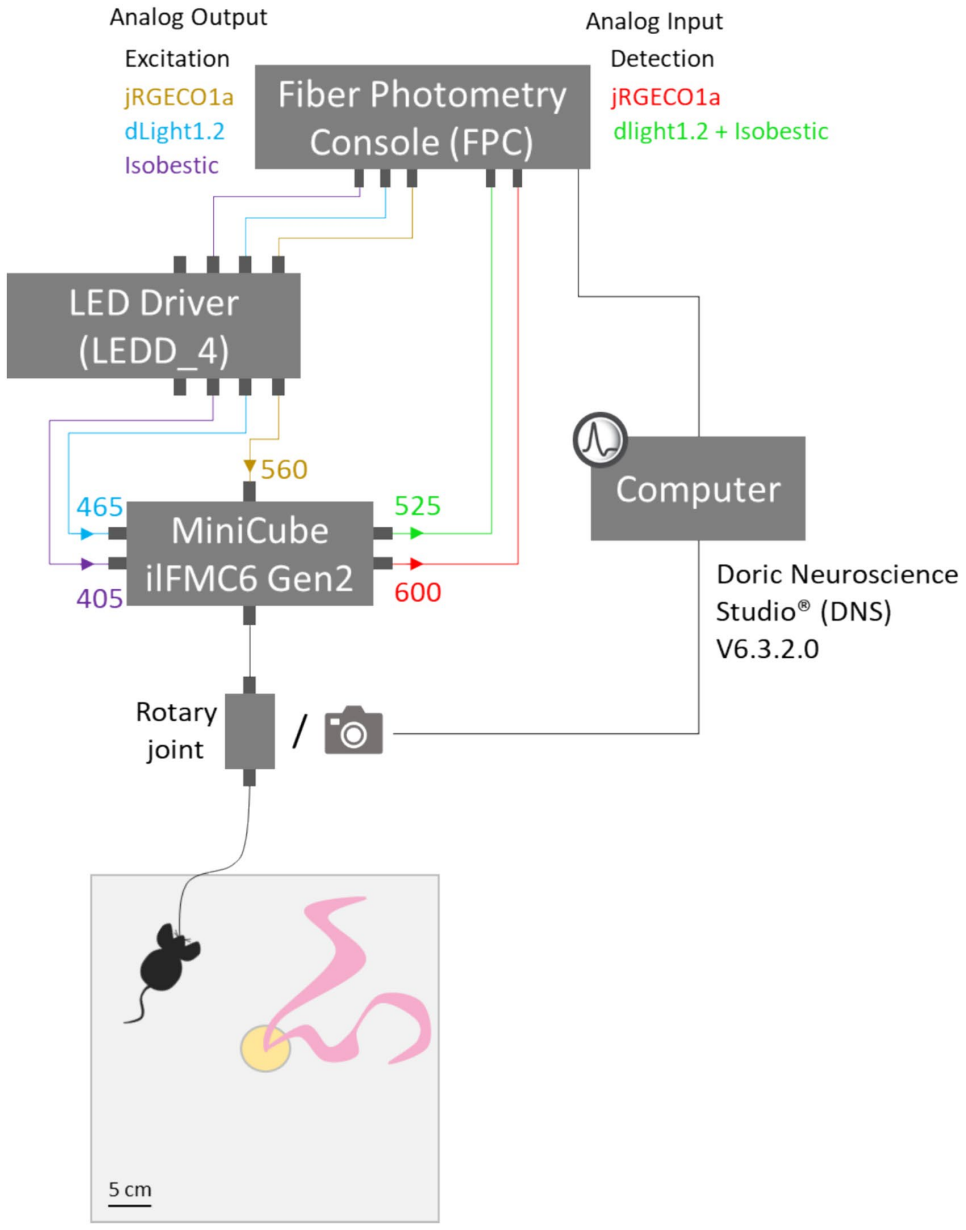
Schematic of the dual-color fiber photometry setup. The system uses LEDs at 405 nm (isosbestic control), 465 nm (dLight1.2 excitation), and 560 nm (jRGECO1a excitation) wavelengths. Fluorescence emissions were detected at 525 nm (green channel for dLight1.2) and 600 nm (red channel for jRGECO1a). The MiniCube with dichroic mirrors and emission filters separates the excitation and emission paths to reduce cross-talk.

Fiber photometry recordings were performed using Fiber Photometry Systems from Doric Lenses® as described in Materials and Equipment and in Figure 2. Three excitation wavelengths 465 nm (dopamine-dependent signal), 405 nm (isosbestic signal), and 565 nm (calcium-dependent signal) were emitted from LEDs (465 nm: LEDC1-B_FC, 405 nm: LEDC1-405_FC, 565 nm: Doric Lenses®), controlled by programmable LED drivers (LED Driver, 4 Channel Model, Doric Lenses®) and transmitted via 0.39 NA, Ø400 μm core multimode prebleached patch cables through a Dual Fluorescence MiniCube (ilFMC6-G2_IE (410–420)_E1 (460–490)_F1 (500–540)_E2 (555–570)_F2 (580–680)_S Fluorescence MiniCube – 6 ports with 2 integrated photodetectors and 3 integrated LEDs, Doric Lenses®). Light intensity at the end of the patch was maintained at 50 μW across sessions, with the average measured power at the fiber tip around 30 to 40 μW. dLight1.2 (dopamine biosensor), jRGECO1a (calcium biosensor) and isosbestic fluorescence wavelengths were measured using femtowatt photoreceivers (Newport, 2,151). The Doric software and LED driver controlled and modulated the excitation lights (465 nm at 209 Hz, 405 nm at 331 Hz, and 565 nm at 251 Hz). Video recordings were made using a camera positioned next to the rotary joint (AFRJ_2×2_PT_400–0.57 Assisted 2×2 Fiber-optic Rotary Joint, 400 μm NA0.57 fiber, 1 m input, optimized for fiber photometry). Video recordings and fiber photometry set-up (camera, Fiber Photometry Console, and LED Driver) were started simultaneously using the “start all devices” option in Doric Neuroscience Studio® (DNS V6.3.2.0), once the mouse has been connected in its home cage. Indeed, the latest version enables the automatic synchronization of recordings by starting and stopping all devices simultaneously. This synchronization can also be verified afterward by examining the timescale in the Doric file (signal recordings).

### 3.6 Histology

A few days after recordings, mice were sacrificed using a ketamine\xylazine overdose (300 mg/kg and 20–30 mg/kg respectively) and then intracardiac perfusion of 4% formalin diluted in PBS (30 mL) at a perfusion rate of 2 mL/min using a peristaltic pump was performed. Brains were dissected, post fixed in 4% formalin diluted in PBS for 4 h at 4°C, and then immersed in a sucrose solution (20% in PBS) overnight. Brains were finally frozen during 20 s at −48°C ± 2, and then stored at −20°C before serial sectioning with a cryostat (14 μm thick 304 μm intervals between samples). The sections were then simply immersed in PBS for 10 min and cover-slipped in Vectashield (Eurobio Scientific) before analysis. The presence of the viruses was analyzed on 18 sections covering the antero-posterior axis of the OT (5.42 mm), using ZEISS® microscope.

### 3.7 Data analysis

#### 3.7.1 Analysis of video recordings

**Figure 3:**
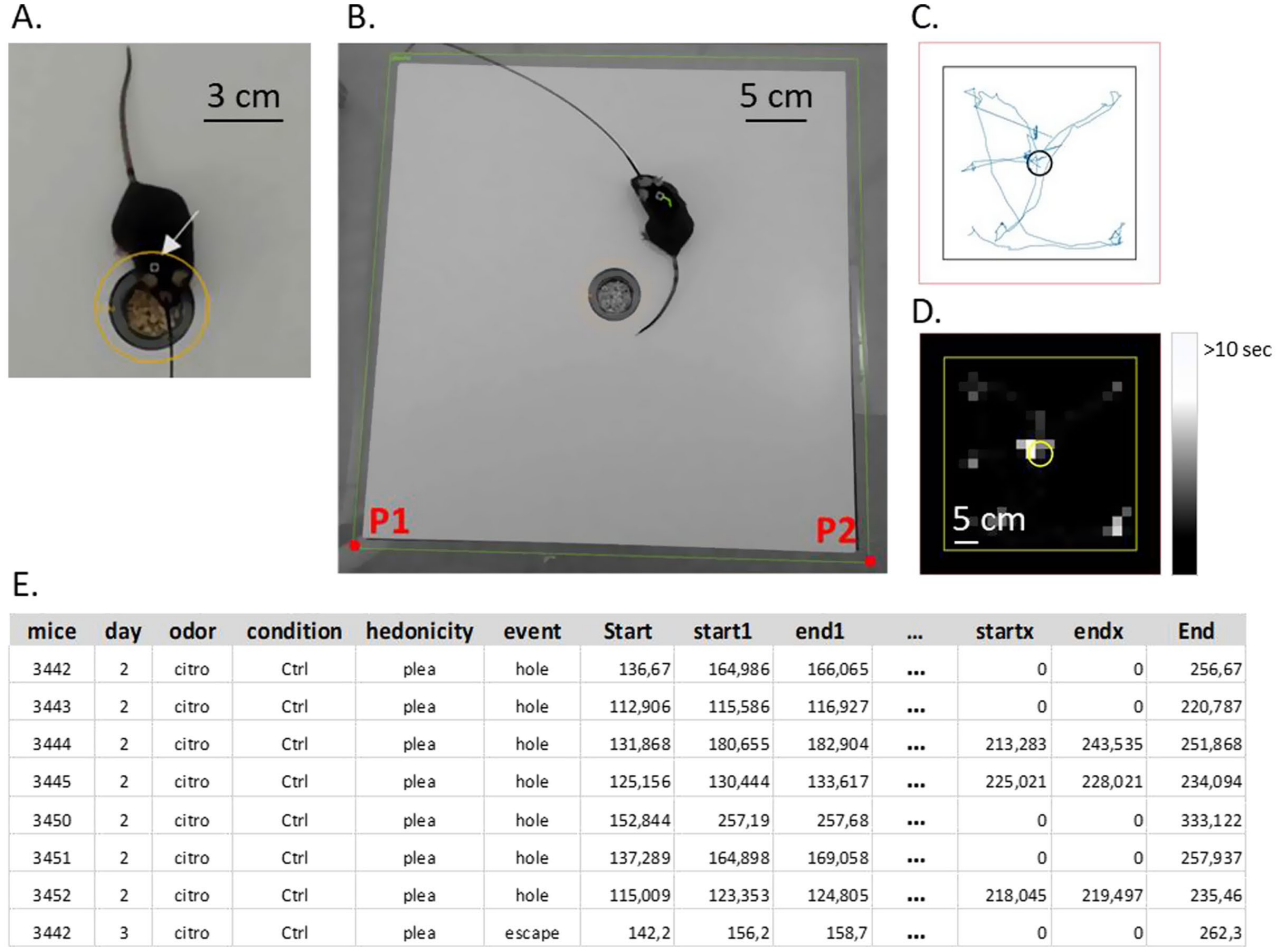
Video recordings and analysis. **(A)** The mouse was detected in the hole. **(B)** Photo of the odor preference test apparatus (hole board 30×30 cm). **(C)** Mouse track map. **(D)** Mouse presence map. **(E)** Example of an Excel spreadsheet that was recalled in the MATLAB program. Citro, citronellol odorant; plea, pleasant; Ctrl, control condition. “start” and “end” correspond to the end and the beginning of the test. “start1” and “end1” corresponds to the beginning and the end of an event of interest.

**Figure 4:**
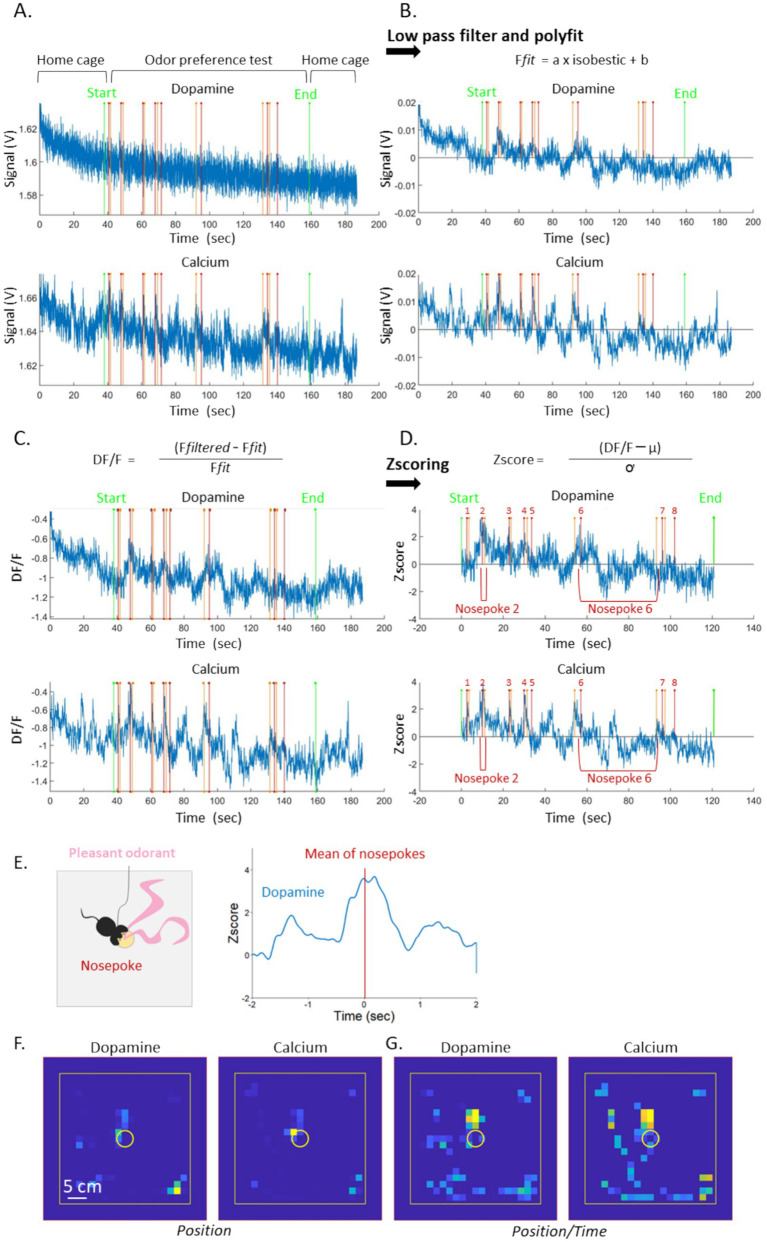
Fiber photometry and video analysis protocol. **(A)** Raw dopamine signal (up) and calcium signal (bottom) during the entire recorded period. Start and End green lines stand for the beginning and the end of the behavioral trial (2 min). Red and orange lines stand for the beginning and the end of each event, respectively, (“*olfactory investigation”*). **(B)** Signals after low pass filter and regression. **(C)** Signals after DF/F calculation. **(D)** Signals after Zscoring (in this example 8 events total from “1” to “8” have been observed). **(E)** Example of averaged dopamine signal for the “*olfactory investigation events*” of one mouse. Timepoint 0 indicates the onset of averaged odorant investigation. **(F)** Dopamine and calcium signals mapped to mouse location on the board. **(G)** Dopamine and calcium signals mapped to mouse location, normalized by the time spent at each location. *μ* and ơ, respectively the mean signal and standard deviation; Ffit, fitted signal.

We analyzed the videos using A2V Volcano, which enabled the detection and tracking of behaving mice. We defined two distinct regions for analysis: the hole (Figure 3A) and the entire board (Figure 3B). To ensure alignment across all videos, we placed two reference points (P1 and P2; Figure 3B) at the bottom corners of the board regions. Mice were detected using pixel analysis and tracked for 2 min while exploring the board (Figures 3C,D). During the 2 min of testing, we identified and referenced times when the mouse entered and left the odorized hole (respectively red and orange lines; Figure 4) as a specific event (“*olfactory investigation events”*) in an Excel spreadsheet (example in Figure 3E). In this spreadsheet, items “start” and “end” represent the beginning and the end of the behavioral test; “start1” and “end1” are the beginning and the end of one event. The spreadsheet is used in the MATLAB® interface to analyze the fiber photometry signal corresponding to the behavior of interest (“*olfactory investigation events*”) (Figure 3E). This allows also to calculate the total duration of the odorized hole exploration.

#### 3.7.2 Fiber photometry data

We pre-processed signals (465 nm, 405 nm and 565 nm) using MATLAB® (Figure 4A). For a detailed description of the data processing with the MATLAB program, see Supplementary materials 1, 2, respectively the code and its detailed instructions. The excel spreadsheet is recalled in MATLAB which then encodes and displays the start (red lines, indTpsStart0) and the end (orange lines, indTpsEnd0) of the event (“*olfactory investigation events*”) (Figure 4). We first applied a standard low-pass Butterworth filter (10 Hz) to smooth the raw fiber photometry signals throughout the entire recording period (Figure 4B). We linearly regressed the filtered dopamine and calcium signals against the isosbestic control signal (405 nm) to model and remove non-neuronal fluctuations (Formozov et al., 2023). Specifically, we fit the model (F*fit* = a x isobestic + b), then used the fitted signal F*fit* to compute DF/F (DF/F = (F*filtered* – F*fit*)/F*fit*) (Figure 4C). This approach corrects for motion artifacts and isolates activity-related fluorescence changes. Additionally, our MATLAB code allows flexible adjustment of both filtering and regression parameter (in the presence of predefined baseline for instance), making preprocessing fully customizable.

Subsequently, the Zscore was computed for the entire signal using MATLAB function following the formula: Zscore = (DF/F – *μ*)/ ơ (Figure 4D). To account for variability due to fiber implantation or recording issues, relative measures such as percent changes or changes in Zscore can be employed. This approach facilitates comparisons between recording sessions or across different animals. It is important to note that we normalized the signal over the entire recording duration instead of using a pre-stimulus baseline due to the variability of self-paced odor exploration, which is further examined in the discussion section.

**Figure 5:**
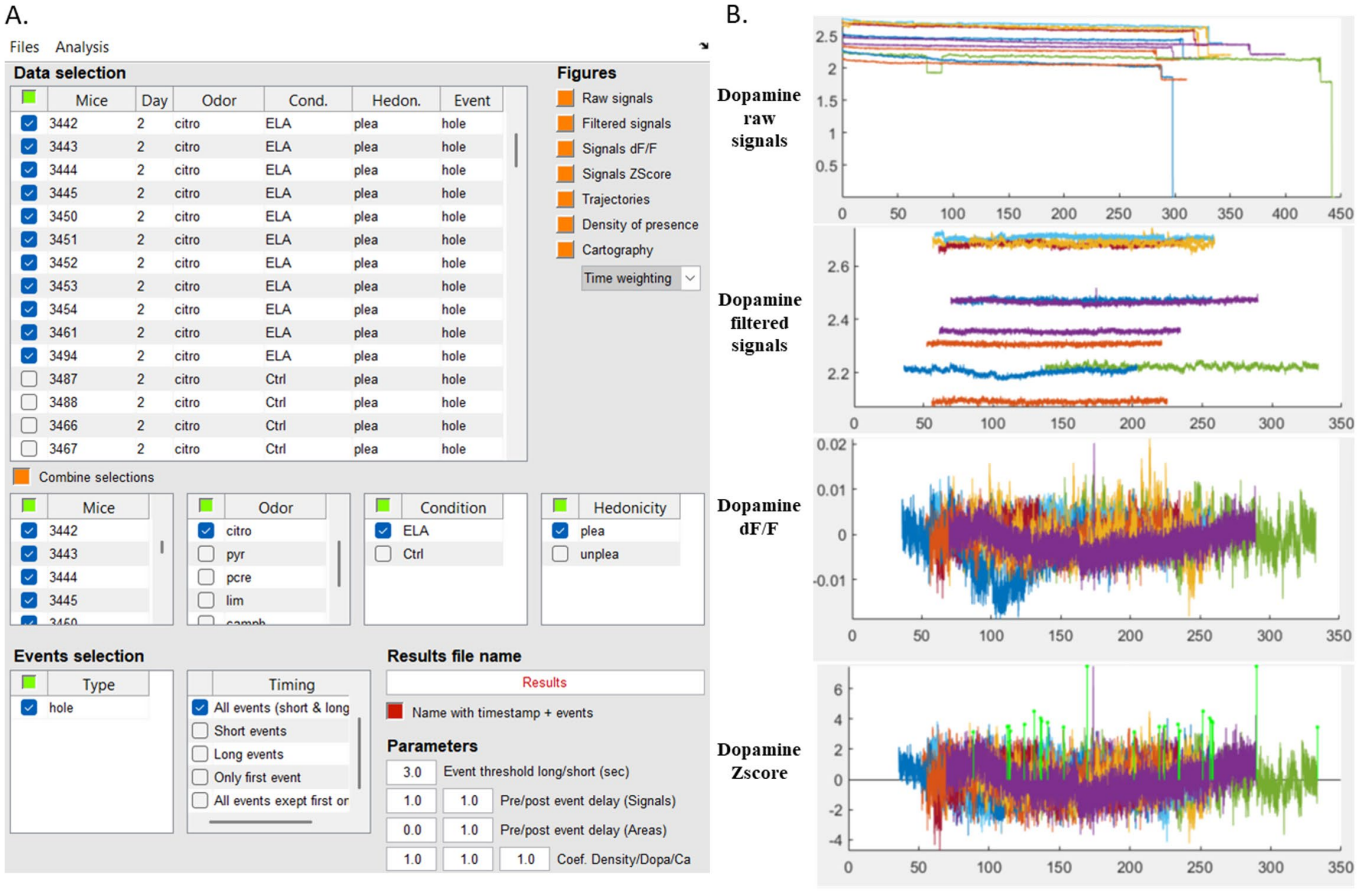
Interface for fiber photometry data navigation and analysis. **(A)** MATLAB-based graphical interface displaying fiber photometry signals synchronized with behavioral events. An example is shown with the selection of data from 11 mice from control (Ctrl) or Stress (ELA) groups investigating citronellol (“citro”) odorant. **(B)** Simultaneous visualization of the dopamine signals corresponding to the selected data and pre-processing before extraction of events of interest. Each colored trace represents the raw, filtered, DF/F or Zscore signal from a single mouse, displayed from the top to bottom. Citro, citronellol; Cond, condition; ELA, Early Life Stress; Ctrl, control; Hedon, hedonicity; plea, pleasant; unplea, unpleasant; hole, odorized hole.

To then analyze a specific behavior such as “*olfactory investigation events*,” the appropriate label must be selected in the MATLAB interface (“hole” for odorized hole selected in the “Type” section, Figure 5). Then selected data are time-aligned to the onset of the event (time point 0), and Z-scores are averaged relative to this reference point. An example of this alignment is shown in Figure 4E, illustrating the dopamine signal from a single mouse. This can be performed across all animals in a given group (e.g., exposed to pleasant or unpleasant odors), with the possibility of separating data by condition, odor type, or other experimental factors. We finally calculated the area under the curve (AUC) for the dopamine and calcium signals before the onset of the event (−0.6 to −0.3 s) and during (from timepoint 0 when entering into the odorized hole zone to +0.3 s) and performed unilateral paired *t*-tests for comparisons. The duration of the analysis window (300 ms) was defined according to the duration of one sniff (Rinberg et al., 2006). We also generated heat maps by combining trajectory data with photometry signals (Figures 4F,G). These maps depict calcium and dopaminergic signals based on mouse location (in pixels; Figure 4F) and can be further normalized by the time spent at each location (Figure 4G; Supplementary materials 1, 2). To determine the appropriate statistical tests, we assessed data normality using the Shapiro–Wilk test. We used parametric tests for normally distributed data, while we applied non-parametric ones when normality assumptions were not met, ensuring robust and appropriate comparisons.

We performed all analysis thanks to data navigation into the MATLAB-based graphical interface displaying the data of the excel spreadsheet (Figure 5). The interface allows for real-time selection of specific parameters for data visualization and analysis. It is an interactive tool for applying filters, normalizing data, extracting DF/F and Zscore values selecting events for targeted analysis of neural activity during exploratory behavior and constructing corresponding heat maps. All plots presented so far are generated using the MATLAB interface. Moreover, we extracted data into Excel spreadsheet with this workflow, enabling further analysis and the generation of refined graphical outputs using RStudio.

## 4 Results

### 4.1 Dopaminergic transmission in the medial olfactory tubercle (mOT) reflected the perception of attractive *versus* unattractive odorants in mice

**Figure 6:**
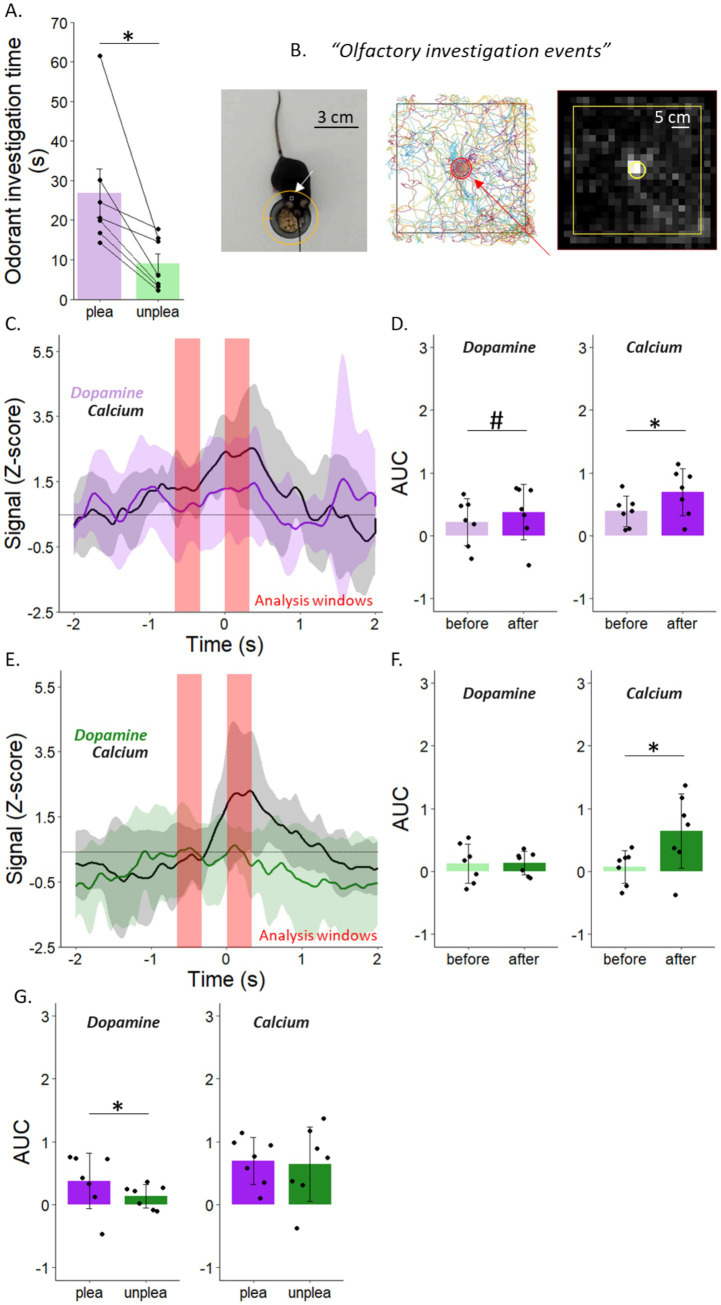
Dopaminergic release in the mOT of adult male mice is driven by the hedonic value of odorant. **(A)** Mice spent significantly more time investigating attractive odorants compared to unattractive ones. **(B)** The mouse was detected on the hole corresponding to “*olfactory investigation events*” (left), full detection enables heatmap representation showing the average exploration pattern on the one-hole board apparatus (right). **(C)** Dopamine and calcium signals during the investigation of the attractive odorant. **(D)** The Area under the curve (AUC) for dopamine (left) and calcium (right) before and during the investigation of the attractive odorant based on the analysis windows around timepoint0 (in red). Calcium signal was higher prior to the nose pock compared to during odor investigation while a similar tendency was observed for dopamine signal. **(E)** Dopamine and calcium signals during the investigation of the least attractive odorant. **(F)** The Area under the curve (AUC) for dopamine (left) and calcium (right) before and during the investigation of the least attractive odorant based on the analysis windows around timepoint0 (in red). While dopamine signal remained unchanged, calcium signal was significantly higher during odorant investigation compared to before. **(G)** The Area under the curve (AUC) for dopamine signal was significantly higher during the investigation of the attractive odorant compared to the least attractive one (left). No difference was observed in calcium signal between the investigation of attractive compared to unattractive odorants (right). Points represent individual data ± sem. *p < 0.05, # *p* = 0.07.

First, from the six odorants tested (three pleasant, three unpleasant), we selected each mouse’s most investigated pleasant odorant and least investigated unpleasant odorant. We first found that mice spent significantly more time investigating the attractive odorants compared to the unattractive ones (unilateral paired *t*-test, *p* = 0.015; Figures 6A,B).

For the fiber photometry results, we first found that both attractive and unattractive odorants elicit higher calcium response during nose pokes compared to prior stimulation period (unilateral paired *t*-test, *p* = 0.01 for attractive odorants and *p* = 0.02 for unattractive ones; Figures 6D,F right). There was no significant difference in dopamine signal before versus during the investigation of unattractive odorants (unilateral paired *t*-test, *p* = 0.4; Figure 6F left). In contrast, dopamine signal tends to be higher during the investigation of attractive odorants compared to prior stimulation period (unilateral paired *t*-test, *p* = 0.07; Figure 6D left).

We finally observed a higher area under the curve of the dopaminergic signal during the investigation of attractive versus unattractive odorants (bilateral paired *t*-test, *p* = 0.041; Figure 6G left). However, we found no significant difference in calcium signaling between the AUCs of attractive and unattractive odorants (bilateral paired *t*-test, *p* = 0.8; Figure 6G right).

### 4.2 Visualization of dopaminergic and calcium signals in response to the escape behavior

**Figure 7:**
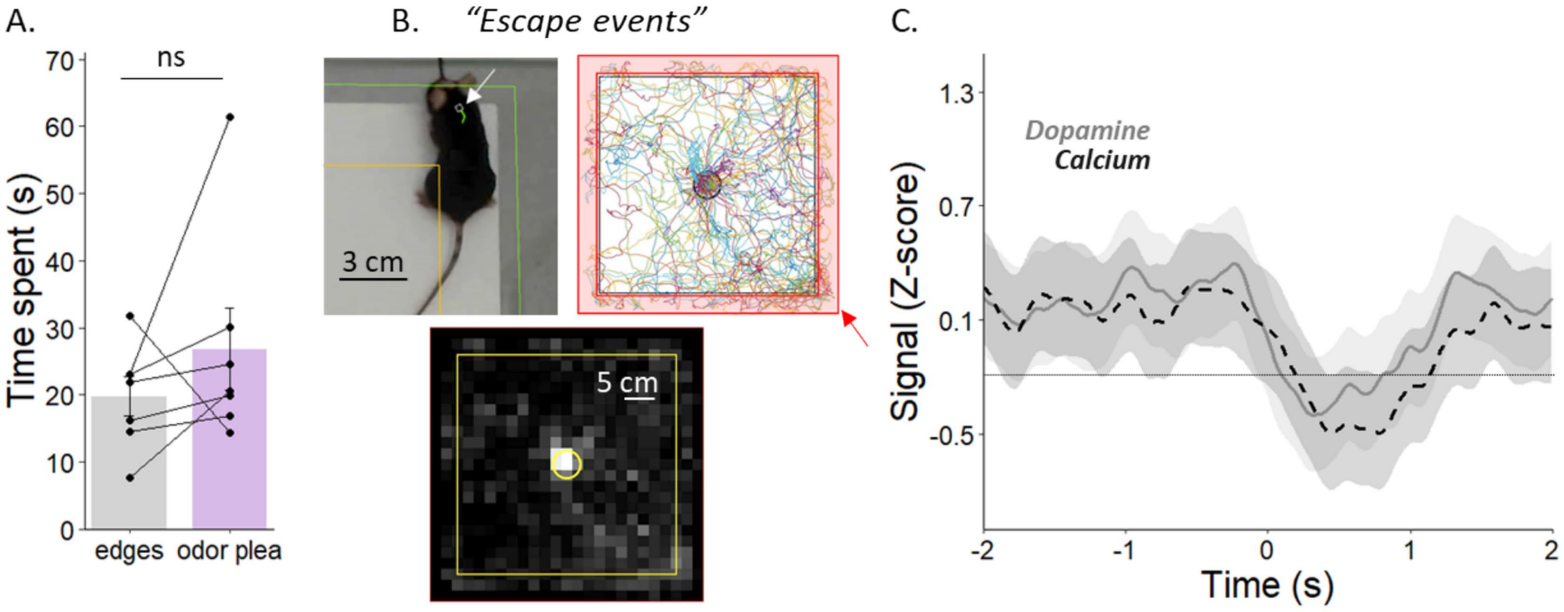
Signals for “*escape events*” show a different pattern compared to the one for “*olfactory investigation events.”* **(A)** The investigation time spent on the edges of the board was similar to that spent on the hole containing the pleasant odorant. **(B)** Mouse detected at the edges of the board, with the head outside the board, corresponds to “*escape events*.” **(C)** Dopamine and calcium signals (Zscore) during the investigation of the edges of the board. Points represent individual data ± sem.

Using our workflow, it is possible to retroactively extract and analyze another type of event. To illustrate this capability, we represented here the exploration of the edges of the board corresponding to an escape behavior (“*escape events”*) (Figures 7A,B). During the 2 min of testing, we identified and referenced the times when the mouse entered and left the board’s edges as a specific event (“*escape events”*) in the same Excel spreadsheet. First, we calculated the total duration spent at the board’s edge and we observed that mice spent the same amount of time investigating the edge of the board compared to attractive odorant for instance (bilateral paired Wilcoxon-test, *p* = 0.21; Figure 7A). Then, in the MATLAB program, we chose to visualize the calcium and dopamine signals accordingly to this specific type of events the same way as “hole” was selected in the “Type” section (Figure 4). We then represented the signals and observed despite similar level of exploration, patterns of calcium and dopamine release during exploration of the edges of the board that were not the pattern we observed in response to odorants (Figure 7C).

**Figure 8:**
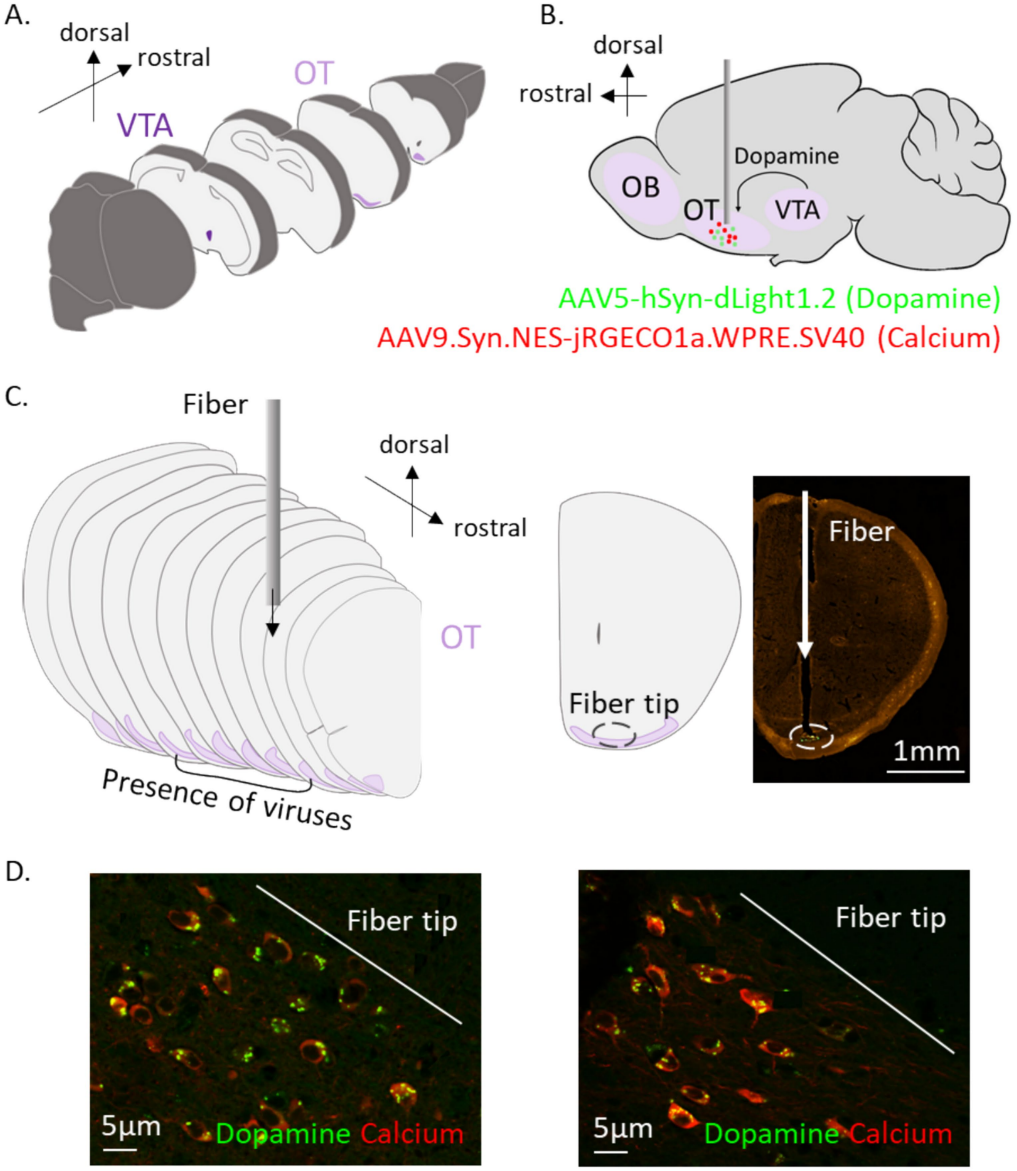
Injection sites, fiber placements, and fluorescence expression in the targeted brain region. **(A)** Representation of the localizations of the OT and the VTA in the mouse brain (exploded view). **(B)** Representation of fiber position and viral expressions. **(C)** Viruses were both expressed in a mean of 6 sections of 14 μm (5.7 mm) of the mOT. **(D)** Photos of cells in the mOT expressing both viruses (x40, ZEISS® microscope).

We finally verified the viral injection and proper implantation of the optical fibers in the animals (Figure 8). We confirmed the virus’s presence on 6 sections ±1 on average, along the antero-posterior axis of the OT; corresponding to 1.604 mm (± 820).

## 5 Discussion

We presented a workflow for using fiber photometry to capture calcium (jRGECO1a) and dopamine (dLight1.2) signals during odor preference tests which assess the spontaneous attraction and exploration of the mice to the odorants. We showed increased level of dopamine in the mOT of mice specifically when investigating attractive odorants compared to unattractive ones. This result demonstrated that a pleasant odorant can act as a natural reward and elicit dopamine release in the mOT. It aligns with previous findings showing that pleasant odorants generate attraction through the recruitment of the VTA (Midroit et al., 2021). However, significant and similar increase of calcium discharge was elicited by odorants of different hedonic values. The area under the curve measures the total signal amplitude over a given period, capturing the overall accumulation of activity. In conclusion, we provided valuable insight into the neural basis of olfactory hedonics, revealing that attractive odorants not only activate the mOT, but also trigger dopamine release in the structure.

We normalized here the signal over the entire recording duration rather than using a conventional pre-stimulus baseline. Standard fiber photometry paradigms often involve longer recording sessions with well-defined stimulation timing, allowing for clear pre- and post-stimulus comparisons. However, our experimental design relies on a two-minute free-exploration paradigm, where mice freely interact with the odorized port at their own pace. The absence of a predetermined stimulus onset and the variability in stimulus exploration durations make it challenging to define a meaningful pre-event baseline. Normalizing across the entire session as well as averaging multiple exploration events per animal minimizes random fluctuations and ensures a consistent and unbiased representation of signal dynamics. Additionally, with before/after analysis, we verified that AUC differences between odor hedonics do not result from pre-activation effects. Nevertheless, our MATLAB code includes an option to preprocess signals using a baseline period free of any stimulation making it adaptable to any experimental design.

In that direction, our workflow can also be applied to any behavior that might retrospectively become of interest. In our case, we highlighted the flexibility of our method by analyzing a different event, the “*escape*” behavior, as frequent on the board as “*olfactory investigation of pleasant odorants*.” Interestingly, it was not associated with dopamine nor calcium patterns in the OT. This supports the hypothesis that pleasant odor investigation depends on the OT activity, while escape behavior, which also drives attractiveness, does not. Not only our method allows for modifications to the type of event being analyzed but it also enables for adjustments to the time window of analysis or for selection by the sequence of the event during the test trial (e.g., the first event, last event, or an average of all events). While fiber photometry combined with synchronized video recording is widely used, most studies indeed rely on predefined event markers, limiting *post hoc* adjustments (Bruno et al., 2021). Our workflow allows dynamic segmentation and reclassification of behavioral events, enabling comparisons across multiple conditions without the need for prior stimulus timing constraints. This approach therefore improves fiber photometry analysis by offering higher flexibility and easier data navigation. This is particularly valuable in free-exploration paradigms, where behavioral engagement is self-paced and unpredictable, ensuring a more adaptive and comprehensive analysis of neural activity in complex settings. It also diminishes the need to limit fiber photometry to control experimental designs, predefined external triggers or strong paradigms such as fear conditioning. The coupling between the signal and the behavioral recordings finally allows spatial analysis by tracking neurotransmitter levels based on the animal’s position. It is important to note that these heatmaps are generated using data from an in-house tracking software. However, navigation and analysis of fiber photometry data are fully compatible with outputs from widely used tools such as DeepLabCut in that outputs data files contain the x and y pixel coordinates of the tracked point, indexed by frame number (i.e., time). These coordinates can then be used to extract behavioral metrics temporally aligned with fiber photometry signals. This flexibility allows users to easily align fiber photometry signals with behavioral events from any tracking method.

This method has some additional limitations. For instance, it cannot precisely identify the responses of different neuronal populations within the mOT. One key limitation is the inability to record the activity of individual cells, as the probe detects fluorescence from the surrounding neuronal populations expressing the biosensor. This issue could be addressed by using transgenic animal lines that target specific neuronal subtypes (Wang et al., 2021) and/or cell specific promoters within the virus. Calcium release in response to pleasant odorants may occur in some sub-population of neurons, potentially in response to dopaminergic input from the VTA. Even if the overall calcium signal could reflect the combined activity of multiple neural populations, the response is likely driven by medium spiny neurons, the main population of the mOT which expresses dopamine membrane receptors (D1 and D2) (Bertran-Gonzalez et al., 2008; Xiong and Wesson, 2016).

Additionally, we used a dual channel fiber photometry setup in which the same isobestic signal was used to preprocess both dopamine and calcium signal. Isosbestic excitation wavelengths can vary slightly depending on the sensor variant and optical setup (Formozov et al., 2023; Molina et al., 2019; Simpson et al., 2024). Nevertheless, our choice of excitation wavelength has been validated in this literature to effectively control for non-specific fluorescence fluctuations in our experimental conditions. Although dLight1.2 is primarily excited at 470 nm, minimal cross-excitation at 405 nm could potentially influence the signal. To minimize cross-talk, we applied emission filters and regression-based correction. Nevertheless, residual effects cannot be fully excluded and may slightly affect DF/F calculations. This limitation is inherent to multiplexed fiber photometry approaches. Our current workflow does not incorporate advanced spectral demixing methods, such as those described by Meng et al. (2018), which enable more precise separation of overlapping fluorescence signals and further reduction of cross-talk (Meng et al., 2018). While implementing these techniques would enhance signal specificity and DF/F accuracy it would here require additional calibration and computation.

Overall, further investigation of ecological behaviors, as well as the exploration of diverse behavioral responses with fiber photometry will enhance both technical and theoretical understanding of brain-behavior correlates, with contributions to greater reproducibility across studies. Recent studies have provided detailed protocols and open-source software to address these challenges (Bruno et al., 2021; Conlisk et al., 2023; Murphy et al., 2023) and here we contribute to the effort by presenting a comprehensive protocol for analyzing the relationship between exploratory behaviors, such as olfactory perception, and brain function.

## Data Availability

The raw data supporting the conclusions of this article will be made available by the authors, without undue reservation.
