# Supplementary material for: Optimized workflow for behavior-coupled fiber photometry experiment: improved data navigation and accessibility
Source: Front Neurosci. 2025 Jul 10;19:1601127. doi: 10.3389/fnins.2025.1601127 (PMC12287066; doi:10.3389/fnins.2025.1601127)
Supplement: Supplementary file 2 [file Data_Sheet_2.DOCX]

**Supplementary materials 2 : Program**

**User Manual**

This manual provides detailed guidance on the use of “**Prog_CartoDopa**” MATLAB code providing the graphical user interface (GUI) developed in the main text.

1. **Data File Formats**

For each recording session, the program relies on a set of files :

- **“doric” file (mandatory) :** contains time-series recordings of dopamine and calcium signals. The filename must include the following information :
  - Animal identification number
  - Recording day, labeled as “day” followed by a number
  - Specific condition
  - The “.doric” file extension
- **Files required for trajectory-based analyses (animal position) :**

These files are optional but required for dopamine and calcium spatial mapping. They are generated by the **A2V_ANA** program (video analysis software), but any tracking software can be used as far as generated files follow the same naming convention as the corresponding “.doric” file.

- Protocol definition file : The Excel file must contain a worksheet named **“fiber”** including all trials (i.e., animals, days, conditions) and all “start” and “end” timestamps as illustrated in Figure3E of the main text.
  - The first row contains the column headers in the following order:
    "mice", "day", "condition", “subgroup” followed by "base1", "base2", and "start"; then the sequence of event intervals: "start1", "end1", "start2", "end2", ..., up to "startN", "endN", and finally "end".
  - The interval [base1 - base2] may be used as a reference window (e.g.,baseline).
  - The interval [start - end] defines the full window that encompasses all event periods.
  - Each subsequent row corresponds to a specific recording with time intervals.
  - An optional column named “event” may be added to specify a particular event type.

If any of "condition", or "subgroup" columns is left empty, the corresponding row (i.e., the trial) will not be included in the analysis and will not appear in the data table displayed in the graphical interface.

1. **File Structure and Directory Tree**

Begin by identifying the pathway where all data, the Excel protocol definition file, and if available the common object definition file (with the .regx extension) are stored. Create subfolders corresponding to each recording day (DAY1, DAY2) and containing all data files of that specific recording day, for all animals and all experimental conditions.


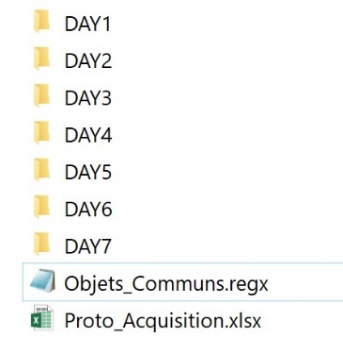

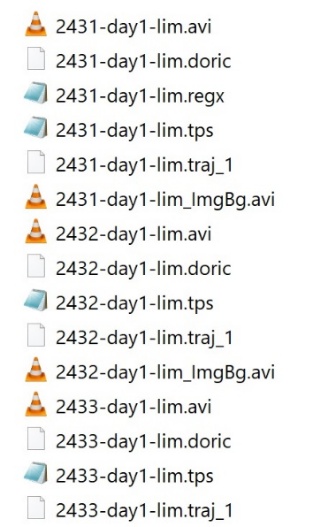
Example of the contents of the “DAY1” subfile:
It typically contains .doric, .traj_1, and .regx files, along with other potential files such as the videos recorded during acquisition, and files generated during video analysis with the A2V_ANA program.

Note that for any given animal (e.g., *“2431”*), **all files must follow the same naming convention**, particularly including the label “day1” to indicate the recording session.

1. **Starting the Program**

To launch the program, open the program files and double-click on **Prog_CartoDopa.m**. This action will open the MATLAB interface and display the program’s source code in the editor window. Run the program so the GUI appears. If the file nomenclature and directory structure have been correctly followed, data loading is straightforward because the entire process is automated.

**Common Object Definition File**

If you are using a common object definition file (board and hole in the case of olfactory preference test), you must first load this file. To do so, select the **File** menu, then choose **Open Common Region File.** For the common file to be recognized and used, it must be loaded before the protocol definition file. Otherwise it will load the file with the same name as the recording.

**Protocol File**

To load a protocol file, select **Open Experiment File (Excel)** under the **File** menu. Selecting the file triggers the loading of all necessary files for analysis, by automatically decoding the various columns from the Excel sheet named **<<** fiber **>>**.

The following files will thus be loaded :

- “**doric**” **data files**: mandatory
- “**traj_1**”**,** “**regx”, and** “**evtx”**: optional

Following this loading process, data validation and preprocessing steps are performed. If some files cannot be read those files will be listed in the **Command Window**, accompanied by an error message.

**Tables in the Graphical Interface**

The main table of the interface “**Data Selection”**, summarizes all the data present in the protocol file. Other tables help filter the trials the user wants to analyze (mice, event, condition, subgroup) and choose the parameters of the processing. The green square buttons at the top of each table allow the user to select or deselect all items at once.

The **Combine Selections** button allows switching between two modes of operation to select the trials for analysis. The choices made in the different secondary tables dynamically update the selection of trials in the main table.

- Button unchecked: selections in the tables are not combined.
- Button checked: selections across tables are combined, meaning that at least one item must be selected in each of the 5 secondary tables; only trials that exactly match the selected items in all five categories will be considered valid in the main table.

**Analysis and Visualization of Raw Data**

Once the data are loaded and selected, preprocessing steps are automatically performed. The different figures can be viewed using the buttons available in the main table. All figures except “**Cartography**” (that needs to be computed aside) can be opened. To close a figure, simply click the corresponding button again.

The figures display the data selected in the main table. If you update the selection, all open figures will update accordingly. If only one trial is selected, the figures also all intervals [start–end]. Intervals are not visible if multiple trials are selected.

Trajectories can only be visualized if the “.regx” (or equivalent) files are loaded. The displayed trajectories correspond to the trials selected in the main table.

The presence density allows to visualize the zones where the animals spent the most time. The shape of the behavioral apparatus (according to “.regx” or equivalent) is discretized using a grid whose cell size can be configured in the MATLAB code. For each grid square, the time spent by each animal is accumulated and then represented with a grayscale gradient. The scaling coefficient **Coef. Density** can be adjusted as follows: if its value is 1, black corresponds to 0 and white to the maximum; on the figure (above the red frame) the maximum value in seconds appears (e.g., max = 45.40 sec). If its value is greater than 1, the image is saturated more to highlight important values; in this case black still corresponds to 0, but white is applied to values greater than a threshold W indicated on the figure (e.g., max = 45.40 sec / W > 9.08 sec).

**Analysis of “Start/End” intervals**

The user must first select the trials in the main table and/or filter them via the secondary tables. Select “Analyze the **Start/End** intervals” in the **Analyses** menu to average the ZScore signals around the event of interests. This is done over the time interval defined in the **Parameters** by choosing the values in **Pre/Post event delay (Signals)** which respectively determine the delay before and after the “Start ”. Figures are automatically generated once calculations are complete. Numerical values are saved in an Excel file whose naming convention is defined via the graphical interface “Naming convention of results files”. The save file corresponds to the pathway where the protocol file is located. The generic name (default “Results” shown in red) can be modified by the user.

**Format of the results file**

This Excel file contains 2 sheets. The first sheet “Zscore+Std” contains the values of the ZScores and standard deviations for dopamine and calcium calculated over the interval (period) ”pre and post signals events”. The first column contains time with a sampling rate corresponding to the acquisition frequency of the signals.

| Time | Dopa_ZS | Dopa_Std | Ca_ZS | Ca_Std |
| --- | --- | --- | --- | --- |
| -1 | 0.285139108 | 1.37848172 | -0.0463101 | 0.88545927 |
| -0.9834 | 0.266349416 | 1.32825554 | -0.0653745 | 0.89959275 |
| -0.9668 | 0.203233184 | 1.23270297 | -0.0798168 | 0.91200591 |
| -0.9502 | 0.127370121 | 1.12986819 | -0.0845018 | 0.92555823 |
| -0.9336 | 0.06796751 | 1.04497423 | -0.0755388 | 0.94249486 |
| … | … | … | … | … |
| 1 | 0.042019211 | 0.94320956 | -0.0199586 | 0.97983058 |

| Data | NumEvt | Dopa_Aire | Ca_Aire |
| --- | --- | --- | --- |
| 2385-7-camphre-Ctrl-plea | 1 | -0.1027175 | 2.15346766 |
| 2385-7-camphre-Ctrl-plea | 2 | 0.16155109 | 1.58273806 |
| 2385-7-camphre-Ctrl-plea | 3 | -0.1520339 | 0.52331873 |
| 2385-7-citro-Ctrl-plea | 6 | 0.49928084 | 0.0399023 |
| … | … | … | … |
| 2385-7-pyr-Ctrl-unplea | 1 | -0.0880622 | 1.96456546 |

The second sheet “Aire” contains the values of the areas under the dopamine and calcium curves calculated for each trial over the interval (period) “pre and post events areas”. The column “NumEvt” corresponds to the number of the “Start/End” event of each trial.

**Mapping**

To calculate and visualize the data in the form of a map, the trajectories and the defined objects files are mandatory. The mapping is calculated on the same principle as the presence density. The space is discretized into pixels in which the ZScores values of dopamine and calcium are summed. For each trajectory, the following operations are performed:

- - The coordinates of each point of the trajectory are retrieved
  - The corresponding square in the discretized space is determined
  - The corresponding start/end period in the ZScores data is determined
  - The dopamine values are averaged over this period
  - The calcium values are averaged over this period
  - These averages are added to the previously obtained averages in the considered square

To carry out all these steps, select the desired trials, then click on the submenu**Calculate Dopa/Ca mapping for selection** in the **Analyses** menu. Once the calculations are finished, you can visualize maps for dopamine and calcium by clicking on the **Cartography** button in the main window.

**Warning:** each time you select new trials in the main list, you must restart the mapping calculation. The display is not updated automatically and you must close and then reopen the visualization windows.

**Calculation options**

Before starting the mapping calculation, you must first choose between the **Ponderation tps** mode and the **Cumul spatial** mode. The first mode allows weighting the results (cumulative dopamine or calcium) by the time spent in each square. The second mode shows the raw cumulative results, without considering the time spent in each square. As with the presence density maps, a scale coefficient can be adjusted to highlight high values. To do this, change the last two values in the fields **Coef. Densité/Dopa/Ca**. To update the display, you do not need to restart the calculations, but you must close and reopen the windows.

Finally, some parameters of the program can be directly modified in the “Flexible parameters” section of the file **Prog_CartoDopa.m**.
